# Altered cardiac rhythm in infants with bronchiolitis and respiratory syncytial virus infection

**DOI:** 10.1186/1471-2334-10-305

**Published:** 2010-10-24

**Authors:** Susanna Esposito, Patrizia Salice, Samantha Bosis, Silvia Ghiglia, Elena Tremolati, Claudia Tagliabue, Laura Gualtieri, Paolo Barbier, Carlotta Galeone, Paola Marchisio, Nicola Principi

**Affiliations:** 1 Department of Maternal and Pediatric Sciences, Università degli Studi di Milano, Fondazione IRCCS Ca' Granda Ospedale Maggiore Policlinico, Milan, Italy; 2 Cardiology Unit, Fondazione IRCCS Ca' Granda Ospedale Maggiore Policlinico, Milan, Italy; 3 Echocardiography Laboratory, IRCCS Centro Cardiologico Monzino, Milan, Italy; 4 Department of Epidemiology, Istituto di Ricerche Farmacologiche Mario Negri, Milan, Italy; 5 Luigi Devoto Department of Occupational Health, Giulio A. Maccacaro Section of Medical Statistics, University of Milan, Milan, Italy

## Abstract

**Background:**

Although the most frequent extra-pulmonary manifestations of respiratory syncytial virus (RSV) infection involve the cardiovascular system, no data regarding heart function in infants with bronchiolitis associated with RSV infection have yet been systematically collected. The aim of this study was to verify the real frequency of heart involvement in patients with bronchiolitis associated with RSV infection, and whether infants with mild or moderate disease also risk heart malfunction.

**Methods:**

A total of 69 otherwise healthy infants aged 1-12 months with bronchiolitis hospitalised in standard wards were enrolled. Pernasal flocked swabs were performed to collect specimens for the detection of RSV by real-time polymerase chain reaction, and a blood sample was drawn to assess troponin I concentrations. On the day of admission, all of the infants underwent 24-hour Holter ECG monitoring and a complete heart evaluation with echocardiography. Patients were re-evaluated by investigators blinded to the etiological and cardiac findings four weeks after enrolment.

**Results:**

Regardless of their clinical presentation, sinoatrial blocks were identified in 26/34 RSV-positive patients (76.5%) and 1/35 RSV-negative patients (2.9%) (p < 0.0001). The blocks recurred more than three times over 24 hours in 25/26 RSV-positive patients (96.2%) and none of the RSV-negative infants. Mean and maximum heart rates were significantly higher in the RSV-positive infants (p < 0.05), as was low-frequency power and the low and high-frequency power ratio (p < 0.05). The blocks were significantly more frequent in the children with an RSV load of ≥100,000 copies/mL than in those with a lower viral load (p < 0.0001). Holter ECG after 28 ± 3 days showed the complete regression of the heart abnormalities.

**Conclusions:**

RSV seems associated with sinoatrial blocks and transient rhythm alterations even when the related respiratory problems are mild or moderate. Further studies are needed to clarify the mechanisms of these rhythm problems and whether they remain asymptomatic and transient even in presence of severe respiratory involvement or chronic underlying disease.

## Background

The most frequent extra-pulmonary manifestations of respiratory syncytial virus (RSV) infection involve the cardiovascular system [1], and include cardiovascolar failure with hypotension and inotrope requirement, associated with myocardial damage, cardiac arrhythmias and pericardial tamponade, particularly in patients admitted to pediatric intensive care units (PICUs) [2-9]. However, the reasons leading to heart involvement during RSV infection are not fully known. As severe bronchiolitis can be associated with pulmonary hypertension [10], it has been thought that the disease itself may lead to right ventricular decompensation with myocardial damage, high cardiac troponin levels and systolic hypotension [11]. Furthermore, it has been demonstrated in other lung diseases, such as bacterial pneumonia, that severe lung involvement can be accompanied by a significant increase in troponin I and T concentrations [12,13] and it is well known that right ventricular strain may precipitate arrhythmias [14]. However, the detection of RSV in myocardial tissue [15,16] and the occurrence of significant pericardial effusion in children with severe RSV bronchiolitis [17-19] suggest that the virus itself may play a direct role in causing heart disease.

As clinically relevant heart problems are usually found in infants whose bronchiolitis is severe enough to require mechanical ventilation [3,6], it is recommended that heart rate and blood pressure should be systematically and carefully monitored in those admitted to PICUs [19], but not in those admitted to semi-intensive or normal pediatric wards. However, no data regarding heart function in infants with bronchiolitis associated with RSV infection have yet been systematically collected although they could throw new light on the pathogenesis of heart involvement during RSV infection and further define the best approach to bronchiolitis.

The aim of this study was to verify the real frequency of heart involvement in patients with bronchiolitis associated with RSV infection, and whether infants with mild or moderate disease also risk heart malfunction.

## Methods

### Study design

This prospective study was carried out at the Department of Maternal and Pediatric Sciences of the University of Milan, Italy, during the winter seasons 2007-2008 and 2008-2009. The protocol was approved by the local Ethics Committee, and written informed consent to study participation was obtained from the patients' parents or legal guardians.

### Study population

The study involved otherwise healthy infants aged 1-12 months who were admitted to hospital because of bronchiolitis during the study period. The exclusion criteria were the presence of a chronic disease increasing the risk of complications of respiratory infection, including chronic disorders of the pulmonary or cardiovascular system, chronic metabolic disease, neoplasms, kidney or liver dysfunction, hemoglobinopathies, immunosuppression, and genetic or neurological disorders. There was no refusal to participate.

Upon admission, the infants' demographic characteristics and medical history were systematically recorded using standardised written questionnaires and, after a complete physical examination, the subjects with a diagnosis of bronchiolitis based on well-established criteria [20] were enrolled. The severity of the disease was defined on the basis of a global evaluation of the signs and symptoms. In particular, on the basis of previously published criteria [20], respiratory illness was considered severe in the presence of all of ≤ 92% pulse oximetry, a respiratory rate of ≥60 breaths/min, marked accessory muscle use, nasal flare or grunting, a heart rate of > 180 beats/min, an inability to feed and a toxic appearance. All of the patients underwent chest radiography, and pneumonia was defined on the basis of the presence of a reticular-nodular infiltrate, segmental or lobar consolidation, or bilateral consolidation [21].

Upon enrolment, Virocult (Medical Wire and Equipment, Corsham, UK) nasopharyngeal swabs were used to collect specimens for the detection of RSV, and a blood sample was drawn to assess troponin I concentrations. On the basis of our previous experience in children with bronchiolitis in which we showed that RSV was the main cause of acute episodes in hospitalized children [22], in this study only RSV was searched on nasopharyngeal secretions. Finally, on the day of admission, all of the infants underwent 24-hour Holter ECG monitoring and a complete heart evaluation with echocardiography. It was decided to estimate pulmonary pressure as well as signs of pulmonary hypertension only in presence of pathologic findings at echocardiography.

During their hospital stay, the infants' clinical signs and symptoms were monitored daily. They were treated with oxygen when saturation was ≤ 95%, and received inhalatory bronchodilators, steroids, antibiotics, intravenous fluids and chest physiotherapy on the basis of the judgement of the attending pediatrician. They were discharged when they were able to maintain > 95% oxymetry without oxygen, but their parents were asked to bring them immediately to the study centre if there were any recurrent or worsening signs and symptoms.

The medical history, general physical condition and clinical symptoms of each patient were re-evaluated by investigators blinded to the etiological and cardiac findings four weeks after enrolment. During this follow-up visit, the patients' history of respiratory tract infections was carefully assessed and 24-hour Holter ECG monitoring 24 hours was repeated.

### Identification of RSV virus

The Virocult nasopharyngeal swabs were tested by means of previously described real-time polymerase chain reaction (PCR) for RSV types A and B [21-24], with total nucleic acids being routinely isolated at the MagnaPureLC Isolation Station (Roche Applied Science, Penzberg, Germany). A universal internal control virus (phocine distemper virus, PDV) was used to monitor the whole process from nucleic acid isolation to real-time detection. The in-house real-time PCRs for RSV and PDV were designed using primer express software (Applied Biosystem, Nieuwerkerk a/d Ijssel, The Netherlands).

RNA was amplified in a single tube, two-step reaction using Taqman reverse transcriptase and PCR core reagent kits (Applied Biosystems, Foster City, CA, USA) and an ABI 7700 or ABI 7500 sequence detection system (Applied Biosystems). A cultured positive control virus was used for each assay. On the basis of proficiency testing data, the sensitivity of each assay was estimated to be less than 500 copies ⁄ mL.

### Evaluation of myocardial damage

To evaluate myocardial damage, serum troponin I levels were measured using the Abbott AxSYM system (Abbott Laboratories, Mississauga, Ontario, Canada) at the time of hospital admission, and were considered indicative of myocardial damage when they were > 1.2 μg/L. The measurements had a coefficient of variation of 10%, and the lower detection limit was 0.3 μg/L.

### Holter ECG monitoring

Three-channel Holter monitors (ElaMedical Spider View 3 channel recorders, Le Plessis-Robinson, France) were positioned immediately after hospital admission, and 24-hour recordings were obtained. After the skin had been prepared, the electrodes were placed to record leads II, V1 and V5; a 1 mV calibration signal was also recorded. The built-in clock started after the electrodes had been attached.

A commercial Holter analysis software (SyneScope, Elamedical, Sorin Group, Le Plessis-Robinson, France) was used to analyse rhythm and heart rate variability (HRV, time-, frequency- and geometric-domain indices) from the Holter tapes. QRS was detected using a level detector, but was manually over-read by a physician. All of the tapes were edited in order to assure the accuracy of the QRS classification. Ectopic beats, noisy data, and artifacts were manually identified and excluded from the HRV analysis. Non-stationarities were avoided by means of trigger adjustment. Average hourly heart rates were determined from the computerised Holter scanner, and maximum, minimum, and mean 24-hour heart rates (with standard deviations, SDs) were calculated for each subject.

The time-domain parameters measured from the Holter tapes were: 1) the average of all normal-to-normal beats (mean NN interval) (mean heart rate); 2) the SD of all NN intervals (SDNN); 3) the SD of the average of NN intervals in all 5-minute segments of the 24-hour recording (SDANN); 4) the mean of the standard deviation in all 5-minute segments of the 24-hour recording (ASDNN); 5) the square root of the mean of the squares of the differences between adjacent NN intervals (rMSSD); and 6) the percentage of > 50 msec differences between adjacent NN intervals. Frequency-domain heart rate variability was also determined, including low-frequency power (LF, total NN interval spectral power between 0.04 and 0.15 Hz), high-frequency power (HF, total interval spectral power between 0.15 and 0.4 Hz), and the LF/HF ratio [25].

### Echocardiographic studies

The echocardiographic studies were made using a real-time ultrasound imaging system system (Acuson Sequoia 512) equipped with 3-, 5-, 7 and 10A, MHz transducers. The echocardiographic measurements were made using standard techniques [26].

M-mode measurements were made in accordance with the recommendations of the Cornmittee of M-Mode Standardization of the American Society of Echocardiography [27], and were used to determine right ventricular internal dimension in diastole (RVID) and left ventricular internal dimensions in diastole (LVID) and systole (LVIS). Left ventricular function was assessed by calculating the percentage fractional shortening of the internal dimension and ejection fraction using standard formulas. Left ventricular mass was also calculated.

The flow velocities across the mitral, tricuspid, aortic and pulmonary valves were recorded from standard pericordial and subcostal positions using pulsed-wave and continuous-wave Doppler transducers.

### Statistical analysis

Continuous variables are given as mean values ± SD, and categorical variables as numbers and percentages. For the comparison between groups (i.e., RSV-positive *vs *RSV-negative), the continuous data were analysed using a two-sided Student's test if they were normally distributed (on the basis of the Shapiro-Wilk statistic) or a two-sided Wilcoxon rank-sum test if they were not. For the comparison within group (i.e., admission *vs *28 ± 3 days after admission in the RSV-positive and RSV-negative groups, separately), the continuous data were analysed using a paired two-sided Student's test or signed-rank test, as appropriate. Categorical data were analysed using contingency table analysis and the chi-square or Fisher's exact test, as appropriate.

## Results

Sixty-nine children with bronchiolitis were enrolled: 34 (49.3%) RSV-positive and 35 (50.7%) RSV-negative. Table 1 shows that there were no differences in gender, age at enrolment, type of delivery, gestational age at birth, birth weight, respiratory problems at birth, respiratory infections or antibiotic courses in the previous three months between the two groups.

**Table 1:** Demographic characteristics of the study population.

Characteristic	RSV- positive n = 34	RSV-negative n = 35	*P *value
Males, No. (%)	18 (52.9)	18 (51.4)	0.90
Mean age at enrolment, days ± SD	142.41 ± 104.8	114.69 ± 108.4	0.22
Type of delivery			
Eutocic, No. (%)	20 (58.8)	20 (57.1)	0.89
Caesarean, No. (%)	14 (41.2)	15 (42.9)	
Gestational age at birth, mean weeks ± SD	37.06 ± 3.63	36.66 ± 3.80	0.66
Birth weight, mean ± SD	2.95 ± 0.86	2.77 ± 0.75	0.34
Respiratory problems at birth, No. (%)	7 (20.6)	8 (22.9)	0.82
Ventilatory support at birth, No. (%)	5 (14.7)	8 (22.9)	0.39
Patients with respiratory infections in previous 3 months, No. (%)	12 (35.3)	8 (22.9)	0.25
Patients treated with antibiotic courses in previous 3 months, No. (%)	6 (17.7)	5 (14.3)	0.70

SD: standard deviation. P-value for comparison between groups, using chi-square or Fisher's exact test, as appropriate, for categorigal data and Student's test or Wilcoxon rank-sum test, as appropriate, for continuous variables.

Table 2 shows the data regarding clinical presentation. Bronchiolitis was mild or moderate in most cases: only three RSV-positive (8.8%) and three RSV-negative patients (8.6%) had severe disease. Disease signs and symptoms, laboratory parameters, radiographic findings, the need for rehydration and oxygen, and the use of antibiotics, steroids, inhalatory bronchodilators, drugs interacting with cardiovascular system and chest physiotherapy were similar in the two groups. None of the patients required PICU admission. None was treated with oral or intravenous bronchodilators. Clinical and echocardiography evaluations showed that the cardiovascular system was always normal, as were cardiac troponin I concentrations.

**Table 2:** Clinical presentation at enrolment.

Characteristic	RSV- positive n = 34	RSV-negative n = 35	*P *value
Severe bronchiolitis, No. (%)	3 (8.8)	3 (8.6)	1.00
Acute onset, No. (%)	6 (17.7)	11 (31.4)	0.18
Rectal temperature ≥38°C, No. (%)	7 (20.6)	6 (17.1)	0.29
Mean temperature ± SD, °C	37.44 ± 0.74	37.02 ± 0.74	0.19
Mean breath frequency ± SD	54.79 ± 14.06	53.73 ± 12.34	0.78
Dyspnea, No. (%)	21 (61.8)	16 (45.7)	0.18
Wheezing, No. (%)	17 (50.0)	16 (45.7)	0.72
Rales, No. (%)	28 (82.3)	29 (82.9)	0.96
Difficulties in feeding, No. (%)	18 (52.9)	12 (34.3)	0.12
Normal clinical heart assessment, No. (%)	34 (100.0)	35 (100.0)	1.00
Normal echocardiographic parameters, No. (%)	34 (100.0)	35 (100.0)	1.00
Mean cTnI ± SD, IU/L	0.009 ± 0.02	0.013 ± 0.02	0.51
Mean CPK ± SD, IU/L	99.27 ± 69.45	79.88 ± 47.32	0.24
Mean LDH ± SD, IU/L	701.96 ± 190.74	597.95 ± 154.17	0.06
Mean SGOT ± SD, IU/L	38.83 ± 10.91	39.57 ± 19.93	0.86
Mean SGPT ± SD, IU/L	23.93 ± 10.98	28.00 ± 14.30	0.22
Pneumonia at X-ray, No. (%)	22 (64.7)	17 (48.6)	0.18
Need for intravenous infusion, No. (%)	15 (44.1)	13 (37.1)	0.56
Need for oxygen therapy, No. (%)	18 (52.9)	21 (60.0)	0.55
Treated with antibiotics, No. (%)	24 (70.6)	21 (60.0)	0.36
Treated with inhalatory bronchodilator, No. (%)	34 (100.0)	35 (100.0)	1.00
Treated with steroids			
Oral steroids, No. (%)	6 (17.6)	5 (14.3)	0.75
Intravenous steroids, No. (%)	2 (5.9)	2 (5.7)	1.00
Treated with drugs interacting with cardiovascular system, No. (%)	0 (0.0)	0 (0.0)	-
Chest physiotherapy, No. (%)	1 (2.9)	2 (5.7)	0.98

SD: standard deviation; cTnI: cardiac troponin I; CPK: creatine phosphokinase; LDH: lactate dehydrogenase; SGOT: serum glutamyl oxaloacetic transaminase; SGPT: serum glutamic pyruvic transaminase. P-value for comparison between groups, using chi-square or Fisher's exact test, as appropriate, for categorigal data and Student's test or Wilcoxon rank-sum test, as appropriate, for continuous variables.

Table 3 summarises the Holter ECG monitoring data. Sinoatrial blocks occurred in 26 RSV-positive patients (76.5%) and only one RSV-negative patient (2.9%) (p < 0.0001). Twenty-five of the 26 RSV-positive patients (96.2%), but not the RSV-negative patient, experienced more than three sinoatrial blocks during the 24 hours, with a maximum of 18 times in one patient. The blocks lasted longer than one second in all cases, and more than two seconds in five (14.7%) (p < 0.05 *vs *RSV-negative patients). Mean and maximum heart rate were significantly higher in the RSV-positive infants (p < 0.05). Among the HRV time-domain parameters, the prevalence of LF periods was significantly higher in the RSV-positive infants (p < 0.05) as was the LF/HF ratio (p < 0.05). Twenty-four hour Holter ECG monitoring 28 ± 3 days later demonstrated the complete regression of the heart abnormalities in all of the RSV-positive infants: no block was recorded and their HRV parameters were similar to those recorded in the RSV-negative patients during the acute phase of the disease and during the convalescent period.

**Table 3:** Heart rate variability in infants with bronchiolitis, by etiology.

Variable	Admission		28 ± 3 days after admission	
	
	RSV-positive (n = 34)	RSV-negative (n = 35)	RSV-positive (n = 34)	RSV-negative (n = 35)
Sinoatrial block, No. (%)	26 (76.5)°^	1 (2.9)	0 (0.0)	0 (0.0)
Sinoatrial block 1-2 sec., No. (%)	21 (61.8)°^	1 (2.9)	0 (0.0)	0 (0.0)
Sinoatrial block > 2 sec., No. (%)	5 (14.7)*"	0 (0.0)	0 (0.0)	0 (0.0)
More than 3 sinoatrial blocks, No. (%)	25 (96.2)°^	0 (0.0)	0 (0.0)	0 (0.0)
Mean heart rate, bpm ± SD	139.23 ± 15.7*"	116.65 ± 15.9	119.37 ± 19.6	112.06 ± 10.1
Maximum heart rate, mean bpm ± SD	204.29 ± 20.6*"	173.30 ± 28.8	179.49 ± 21.8	170.10 ± 21.3
Minimum heart rate, mean bpm ± SD	49.62 ± 17.1	45.61 ± 26.10	49.31 ± 19.45	48.44 ± 20.14
SDNN (ms), mean ± SD	58.34 ± 26.37	54.61 ± 24.11	56.46 ± 22.21	55.39 ± 22.68
SDANN (ms), mean ± SD	49.61 ± 28.75	45.57 ± 25.70	47.66 ± 25.55	46.91 ± 26.96
ASDNN (ms), mean ± SD	37.11 ± 17.20	33.33 ± 13.70	35.64 ± 18.52	34.39 ± 16.31
rMSSD (ms), mean ± SD	20.31 ± 19.55	19.30 ± 16.99	19.25 ± 19.43	19.77 ± 19.03
PNN50, mean % ± SD	4.21 ± 7.2	2.93 ± 4.5	3.33 ± 5.3	3.22 ± 4.9
LF (ms^2^), mean ± SD	636.13 ± 306.7*"	369.48 ± 276.7	373.49 ± 269.73	369.55 ± 288.76
LF (nu), mean ± SD	33.93 ± 6.8*"	24.91 ± 5.6	25.01 ± 5.9	24.99 ± 6.1
HF (ms^2^), mean ± SD	271.06 ± 573.5	106.09 ± 152.5	143.9 ± 155.5	176.73 ± 169.6
HF (nu), mean ± SD	7.10 ± 7.6	6.76 ± 2.8	6.76 ± 4.5	6.85 ± 4.9
LF/HF ratio, mean ± SD	4.78 ± 1.60*"	3.68 ± 1.48	3.69 ± 1.40	3.64 ± 1.55

SD: standard deviation; bpm: beats per minute; SDNN: standard deviation of all NN intervals; SDANN: standard deviation of the average of NN intervals in all 5-minute segments of the 24-h recording; ASDNN: mean of the standard deviation in all 5-minute segments of the 24-h recording; rMSSD: square root of the mean of the squares of the differences between adjacent NN intervals; pNN50: percentage of differences between adjacent NN intervals of > 50 msec; LF: low-frequency power; HF: high-frequency power; nu: normalised units.

°p < 0.0001 and *p < 0.05 for the comparison between groups (i.e., RSV-positive *vs *RSV-negative upon admission); ^p < 0.0001 and "p < 0.05 for the comparison within group (i.e., admission *vs *28 ± 3 days after admission in the RSV-positive group); no other significant difference between or within-group.

Table 4 shows the associations between sinoatrial block and the other variables in the infants with RSV infection. The only variable that was independently associated with the occurrence of sinoatrial block was RSV viral load: blocks were significantly more frequent in the infants with a viral load of ≥100,000 copies/mL than in those with a lower viral load (p < 0.0001). The association remains significant even after excluding patients with severe disease. There was no association between sinoatrial block and gestational age, birth weight, neonatal problems, or the presentation or severity of bronchiolitis.

**Table 4:** Associations between sinoatrial block and different variables in infants with bronchiolitis and RSV infection.

Variable	Patients with sinoatrial block (n = 26)	Patients without sinoatrial block (n = 8)	P
RSV viral load			
< 100.000 cp/mL	4 (15.4)	8 (100.0)	< 0.0001
≥100.000 cp/mL	22 (84.6)	0 (0.0)	
Gestational age			
≥37 weeks	10 (38.5)	4 (50.0)	0.69
< 37 weeks	16 (61.5)	4 (50.0)	
Birth weight			
≥2,500 g	5 (19.2)	2 (25.0)	1.00
< 2,500 g	21 (80.8)	6 (75.0)	
Respiratory problems at birth			
No	21 (80.8)	6 (75.0)	1.00
Yes	5 (19.2)	2 (25.0)	
Ventilatory assistance at birth			
No	23 (88.5)	6 (75.0)	0.57
Yes	3 (11.5)	2 (25.0)	
Severe bronchiolitis			
No	23 (88.5)	8 (100.0)	1.00
Yes	3 (11.5)	0 (0.0)	
Rectal temperature ≥38°C			
No	21 (80.7)	6 (75.0)	0.98
Yes	5 (19.2)	2 (25.0)	
Dyspnea			
No	9 (34.6)	4 (50.0)	0.68
Yes	17 (65.4)	4 (50.0)	
Wheezes			
No	12 (46.1)	5 (62.5)	0.69
Yes	14 (53.9)	3 (37.5)	
Rales			
No	4 (15.4)	2 (25.0)	1.00
Yes	22 (84.6)	6 (75.0)	
Difficulties in feeding			
No	13 (50.0)	3 (37.5)	0.69
Yes	13 (50.0)	5 (62.5)	
Pneumonia at X-ray			
No	8 (30.8)	4 (50.0)	0.41
Yes	18 (69.2)	4 (50.0)	

Percentages in parenthesis. P-value for comparison between groups, using chi-square or Fisher's exact test, as appropriate.

## Discussion

The results of this study indicate that bronchiolitis during the course of RSV infection is frequently associated with sinoatrial blocks, an increase in absolute heart rate, and an increase in the LF component of HRV. All of these findings seem to be specific of RSV infection because they have not been demonstrated in children with bronchiolitis caused by a different infectious agent. Our data confirm and extend what has been previously reported by other authors who have found that RSV infection can be associated with cardiac rhythm alterations [8,9,28].

Sinoatrial blocks are rare in pediatrics but, when symptomatic, have been described in otherwise healthy children and patients with heart malformations or myocarditis [29]. To the best of our knowledge, this is the first report that associates sinoatrial blocks and RSV bronchiolitis. In our study population, the sinoatrial blocks were always asymptomatic and disappeared with recovery from the respiratory disease, thus suggesting that they are reversible. Furthermore, the significantly increased mean heart rates and the high incidence of the LF components of HRV (usually considered a possible marker of cardiac damage) [25,26], were only observed during the acute phase of RSV infection. Moreover, none of the children showed any clinical sign or symptom resembling those described in subjects with symptomatic sinoatrial block, any echocardiographic alteration or any increase in troponin I concentrations. All of these findings support the hypothesis that RSV can specifically alter the electrical conduction system, but that these alterations are benign and transient. Considering that current arrhythmia guidelines do not recommend any kind of intervention in transient sinoatrial block [30], on the basis of our findings we do not recommend routine cardiac monitoring of infants with bronchiolitis in general wards. However, our findings highlight the need of further studies on the impact of sinoatrial blocks in patients with chronic underlying disease at risk of complications during RSV infection.

One limitation of this study is that the population is too small to allow any definite conclusions to be drawn and so further studies of larger series are needed. It seems to be particularly important to study more severe cases in order to verify whether more significant lung involvement can precipitate arrhythmias and cause more serious clinical problems. It is interesting that in our population all the three cases of severe bronchiolitis showed a sinoatrial block. The very low number of subjects with severe infection could have limited the statistical power to detect between group differences according to disease severity. Another limitation is the fact that only RSV has been searched in respiratory secretions. Despite it represents the absolute main cause of bronchiolitis in infants and in various studies it has been detected as single pathogen in more than 60% of the cases [1,22], it could be interesting to understand whether other viruses may cause a similar cardiac involvement as well as sinoatrial blocks could be more severe and persistent when RSV acts as a co-pathogen with another virus. On the basis of our data, it can be hypothesised that RSV infection is one of the possible causes of these alterations and may even be the direct cause in some cases.

Our data support the hypothesis that the heart involvement diagnosed in some cases of bronchiolitis associated with RSV infection [2-8] could be due to direct viral damage of heart tissue or to immunologic mechanisms rather than the lung alterations that follow respiratory infection. In addition to the changes in the heart electrical conduction system, which was exclusively recorded in our RSV-positive patients, this hypothesis is supported by the fact that most of our children had mild or moderate disease, and were therefore presumably free of pulmonary hypertension and the significant lung damage conditioning right heart failure. Furthermore, although the small number of patients prevented the use of multivariate analysis, the close correlation between sinoatrial block and RSV load suggests that RSV could play a direct role in inducing arrhythmia. This association between high viral load in respiratory secretions and prevalence of sinoatrial blocks is intriguing because since the role of viral load in respiratory secretions is controversial several recent studies have highlighted its importance in conditioning respiratory symptoms and disease's severity [31-35].

## Conclusions

RSV seems associated with sinoatrial blocks and rhythm alterations even when the resulting respiratory difficulties are mild or moderate. Further studies are needed to clarify the mechanisms of these rhythm problems and whether they remain asymptomatic and transient even in presence of severe respiratory involvement or chronic underlying disease. Finally, as RSV can cause respiratory illnesses other than bronchiolitis, further researches specifically aimed at defining the relationships between RSV and the heart are urgently needed regardless of the clinical picture.

## List of abbreviations

(mean NN interval): Average of all normal-to-normal beats; (bpm): beats per minute; (cTnI): cardiac troponin I; (CPK): creatine phosphokinase; (HRV): heart rate variability; (HF): high-frequency power; (LDH): lactate dehydrogenase; (LVID): left ventricular internal dimensions in diastole; (LVIS): left ventricular internal dimensions in systole; (LF): low-frequency power; (ASDNN): mean of the standard deviation in all 5-minute segments of the 24-h recording; (nu): normalised units; (PICUs): pediatric infectious disease units; (pNN50): percentage of differences between adjacent NN intervals of > 50 msec; (PDV): phocine distemper virus; (PCR): polymerase chain reaction; (RSV): respiratory syncytial virus; (RVID): right ventricular internal dimension in diastole; (SGOT): serum glutamyl oxaloacetic transaminase; (SGPT): serum glutamic pyruvic transaminase; (rMSSD): square root of the mean of the squares of the differences between adjacent NN intervals; (SD): standard deviation; (SDNN): standard deviation of all NN intervals; (SDANN): standard deviation of the average of NN intervals in all 5-minute segments of the 24-h recording.

## Competing interests

The authors declare that they have no competing interests.

## Authors' contributions

SE and NP designed the study and co-wrote the manuscript.

PS and SG performed the cardiologic studies.

PB assisted in the interpretation of cardiologic data.

SB carried out the real-time PCR.

CT and LG visited the patients during hospitalization.

ET performed the follow-up visits.

CG performed the statistical analysis.

All authors read and approved the final manuscript.

## Pre-publication history

The pre-publication history for this paper can be accessed here:

http://www.biomedcentral.com/1471-2334/10/305/prepub
